# Supplementary figures and images for: Evaluating the kidney disease progression using a comprehensive patient profiling algorithm: A hybrid clustering approach
Source: PLoS One. 2025 Jul 11;20(7):e0310749. doi: 10.1371/journal.pone.0310749 (PMC12250582; doi:10.1371/journal.pone.0310749)

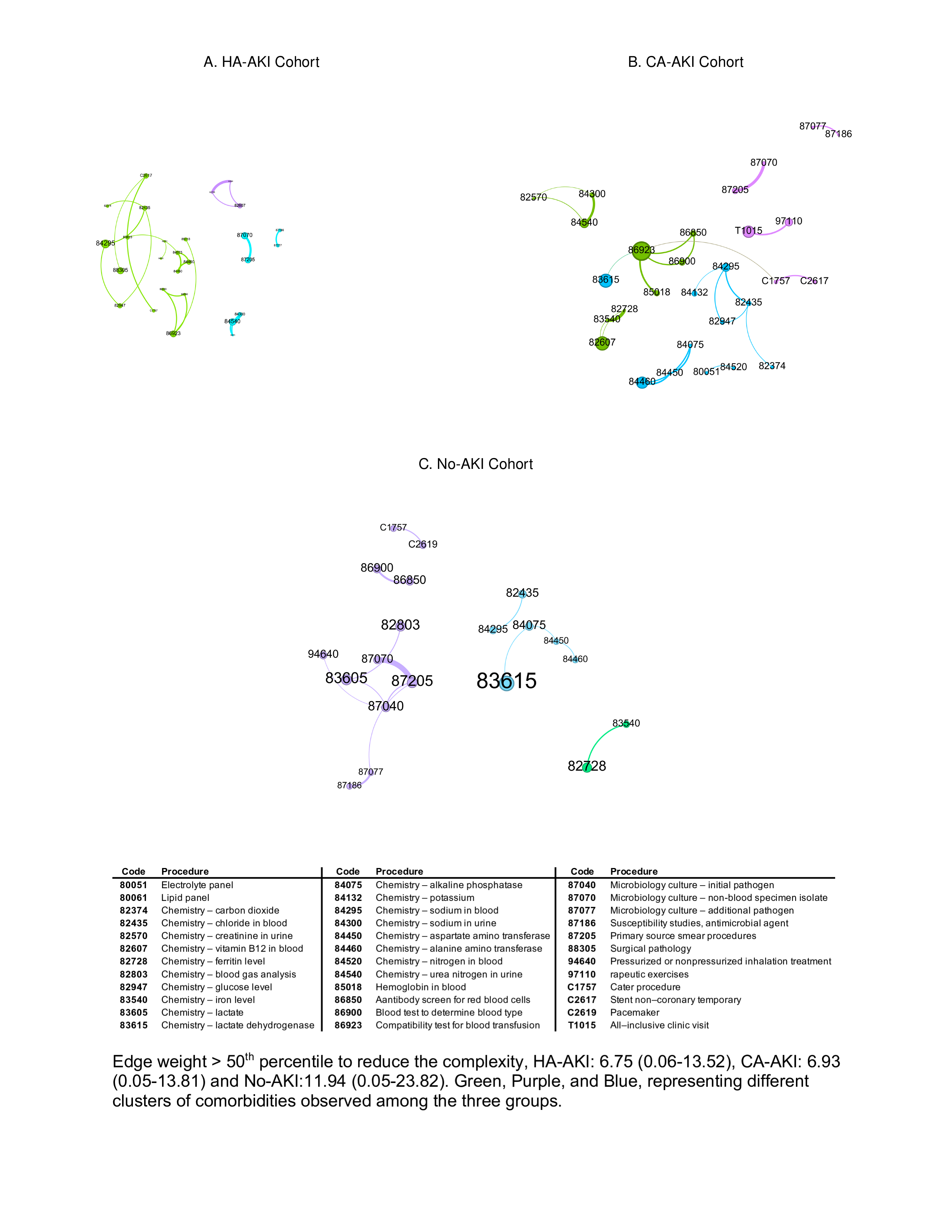

Supplement: S1 Fig — The procedure clusters identified by the community detection algorithms and designated as three colors: green, purple, and blue. Each Node (e.g., 84295, 87070, 84540 etc.) represent procedures, node size indicates prevalence, while edge thickness (i.e., line between two nodes) represents the observed‐to‐expected ratio (OER) (> 90th percentile). (TIFF) [file pone.0310749.s001.tiff]
