# Supplementary material for: Evaluating the kidney disease progression using a comprehensive patient profiling algorithm: A hybrid clustering approach
Source: PLoS One. 2025 Jul 11;20(7):e0310749. doi: 10.1371/journal.pone.0310749 (PMC12250582; doi:10.1371/journal.pone.0310749)
Supplement: S1 File — (DOCX) [file pone.0310749.s006.docx]

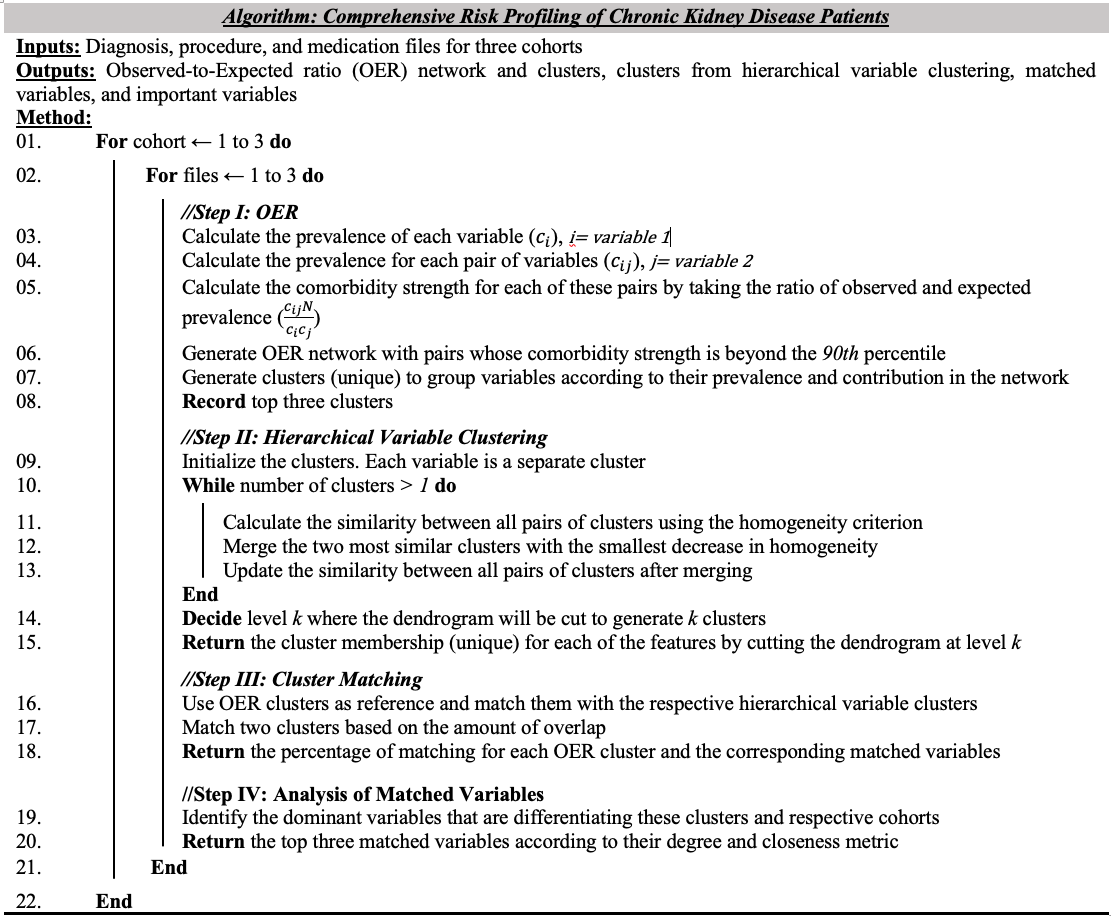


**Hierachical Variable Clustering *ClustOfVar* method:** For quantitative variables, homogeneity is measured by the squared Pearson correlation, while for qualitative variables, it is measured by the correlation ratio. Mathematically, the homogeneity $H\left( C_{k} \right)$ for a cluster *C_k_* is defined as:

$$H\left( C_{k} \right)=\sum_{x_{j}\in C_{k}} r_{x_{j},c_{k}}^{2}+\sum_{y_{j}\in C_{k}} \eta_{c_{k}|y_{j}}^{2}$$

Where $r_{x_{j},c_{k}}^{2}$ is the squared Pearson correlation between quantitative variable $x_{j}$ and the synthetic variable $c_{k}$ and $\eta_{c_{k}|y_{j}}^{2}$ is the correlation ratio between qualitative variable $y_{j}$ and the synthetic variable $c_{k}$. The synthetic variable $c_{k}$ is created using principal component analysis (PCA) for quantitative variables and multiple correspondence analysis (MCA) for qualitative variables. This synthetic variable represents the first principal component derived from applying PCA or MCA to all variables within the cluster. We utilized a hierarchical clustering approach to perform the clustering process**.** This method begins by treating each variable as an individual cluster and iteratively merges clusters to minimize the loss of homogeneity. The dissimilarity between any two clusters $C_{1}$ and $C_{2}$ is defined as:

$$d\left( C1,C2 \right)=H\left( C1 \right)+H\left( C2 \right)-H\left( C1\cup C2 \right)$$

The algorithm continues to merge clusters until all variables are consolidated into a single cluster, resulting in a dendrogram. This dendrogram can be cut at different levels (*k*) to achieve the desired number of clusters. The hierarchical approach provides a clear visual representation of the clustering process, helping to determine the optimal number of clusters.
