# Supplementary material for: Evaluating the kidney disease progression using a comprehensive patient profiling algorithm: A hybrid clustering approach
Source: PLoS One. 2025 Jul 11;20(7):e0310749. doi: 10.1371/journal.pone.0310749 (PMC12250582; doi:10.1371/journal.pone.0310749)
Supplement: Attachment — Submitted filename: Response to Reviewer comments _final.pdf [file pone.0310749.s008.pdf]

RESPONSE TO THE REVIEWER COMMENTS

<u>REVIEWER #1</u>:

The manuscript from Al-Mamun et al aims to characterize the clinical phenotypes of AKI and/or CKD patients, using different clustering and network algorithm on a large electronic health records dataset. Patients with a first diagnosis of CKD were selected and the presence or absence of AKI in the previous three years was characterized. Then, network and clustering of medical records were performed in “hospital acquired AKI”, “community acquired AKI” or “no previous AKI” patients with CKD. The main result is unclear. Despite appropriate method description, it is not clear how to interpret data. The multiplicity of tables, figures, analyses, supplementary files, specific/unusual metrics (with limited commentary) and the overall mixing in figure numbering, missing figure labels and cryptic coded labelling further complicates understanding.

Regarding the study design, it is not very clear what healthcare is provided by WVU, which is highly relevant to understand the selection of patients. Are patients hospitalized or having medical visits? It would also be very interesting to characterize the patient trajectories as in the number of hospital/medical visits during the 3 years prior CKD, and at least eGFR and albuminuria levels observed at CKD diagnosis if available.

AU: We appreciate the reviewer’s insightful comments and recognize the importance of clarifying the healthcare setting from which our patient cohort was derived.

(1) **<u>Clarification of Data Source and Healthcare Setting</u>**: The dataset used in this study is sourced from TriNetX, a research network that aggregates real-world data from multiple hospital systems located in West Virginia, rather than being limited to West Virginia University (WVU). To address potential confusion, we have revised Methods sections 2.1 and 2.2 to explicitly state the source of data and the scope of the healthcare system providing patient records.
(2) **<u>Patient Selection and Healthcare Utilization</u>**: The cohort includes both hospitalized patients and those with outpatient visits, covering inpatient, ambulatory, and emergency department encounters. We acknowledge the importance of characterizing patient trajectories to understand healthcare utilization patterns before CKD diagnosis.
(3) **<u>Clinical Parameters at CKD Diagnosis</u>**: Unfortunately, our dataset does not include serum creatinine values or eGFR at CKD diagnosis, as CKD status was identified using ICD codes rather than laboratory values. Albuminuria data is not available in our dataset, which is a known limitation. We have acknowledged this limitation in the Limitation section” in Discussion section and emphasized the potential value of including these parameters in future studies.

RESPONSE TO THE REVIEWER COMMENTS

The paper could be improved by reducing the number of tables/figures, while improving their quality/readability. Many diagnoses/procedures remain coded by ICD or CPT codes, which limits interpretations. A schematic of representation of the network terms/items and metrics and their interpretation could enhance understanding.

AU: We have reduced the number of figures and tables in the revised version. To improve the readability, we added further details in the footnotes (e.g., description of the ICD and CPT codes) of the figures and tables. We reduced the graph complexity only for visualization purpose by setting the edge weight to 50th percentile. One can change this threshold to evaluate the overall network complexity as per their need. All the changes were highlighted in the highlighted revised version.

Table 1 is confusing as it is not stated that these are actually CKD patients, with a history of AKI or not. Also, the table data is somewhat redundant (NO AKI vs AKI results and p-values could be removed).

AU: Table one reorganized and titles and footnotes were revised to improve the readability.

(1) **Clarification of Patient Population**: The table title and footnotes have been revised to explicitly state that all patients included have CKD, categorized based on their history of AKI (HA-AKI, CA-AKI, or No-AKI). Column headers have been renamed for clarity to clearly distinguish the patient groups.
(2) **Reduction of Redundant Data**: To enhance readability and avoid redundancy, we removed the No-AKI vs. AKI results and p-values, as these were largely repetitive of the individual HA-AKI vs. No-AKI and CA-AKI vs. No-AKI comparisons. The table now focuses on the pairwise comparisons (HA-AKI vs. No-AKI and CA-AKI vs. No-AKI), which better highlights the key differences among groups.
(3) **Improved Formatting for Readability**: The table layout has been adjusted to enhance visual clarity, with clear group separations and improved spacing. Key variables are now organized in a more logical order, grouping related comorbidities and demographic factors together for easier interpretation.

Figure 2a and 2b would be greatly improved by ordering items by the most relevant metrics, such as centrality in No-AKI and labelling the different diagnosis. Cohort's names in the text should be corrected.

AU: We appreciate the reviewer's suggestion to enhance Figure 2a and 2b for improved readability and interpretation. In response:

(1) **Figure Enhancements**: We have reordered items in Figure 2a and 2b based on centrality metrics (degree and betweenness centrality) in the No-AKI cohort to highlight the most relevant findings. Diagnoses in the figures are now explicitly labeled to improve interpretability and eliminate reliance on ICD codes alone.

However, we have also updated and revised the Supp Fig 1 for the procedure networks with descriptions of the procedures as a footnote.

(2) **Text Corrections**: The cohort names have been corrected throughout the manuscript for consistency.

Table 3 provides similarity values, but it is unclear how to interpret the results, and the associated paragraph mainly repeats observation rather than
- What software were used? Was it R? any other?
- Where are figure legends?
- There are MANY issues in figure numbering

AU: We appreciate the reviewer’s insightful comments and have made the following revisions to enhance clarity and improve figure organization.

(1) **Clarification of Table 3 Interpretation**: We have revised the description of Table 3 to clearly define how to interpret similarity values and how betweenness centrality plays a role in network structure and disease progression. The following explanation was added: "Betweenness centrality measures the extent to which a node lies on the shortest paths between other nodes, indicating its influence within the network by controlling the flow of information. Diagnoses with high betweenness centrality act as 'bridges' connecting different clusters or groups of diagnoses, potentially playing a critical role in disease progression or serving as intermediaries in that pathway." We also adjusted the associated paragraph to focus on key insights rather than repeating observations. (line 324-329)

(2) **Software Used & Methodology Transparency**: We explicitly stated in Methods section 2.9 that: "All network analyses, including calculations of degree and betweenness centrality, were performed using R (ClustOfVar package).”

(3) **Figure Legends & Labeling Enhancements**: Figure legends were reviewed and expanded to clearly define node colors and their relevance. Specifically, we now state: "Clusters were identified using three distinct colors: Green, Purple, and Blue, representing different subgroups of comorbidities and procedures."

(4) **Figure Numbering Corrections**: We systematically revised and corrected figure numbering throughout the manuscript and supplementary materials. All cross-references to figures in the text were updated to match the correct numbering sequence.

REVIEWER #2:

This study uses an innovative comprehensive patient profiling tool accurately projects a patient’s progression from AKI to CKD using diagnosis and procedures data. It demonstrates how different comorbidities and procedures can be analyzed using a profiling method to elucidate factors influencing the transition from AKI to CKD. It identifies both similar and distinct risk factor clusters among patient groups and has the potential to uncover non-traditional risk factors for chronic diseases by identifying less prevalent or previously unknown comorbidities and procedures.

However, as described in the Limitations of the study, the retrospective design introduces the risk of missing data, which could lead to confounding bias, and should be better discussed in the manuscript as described below:

1) In Table one, “race and “ethnicity” have a high percentage of “unknown” data. As in CKD we know the importance of this , it should better explore and discussed (why is only “white” and “nonwhite” ?, for example). In discussion is said that “the risk of AKI and its progression to CKD has been associated with factors such as increasing age, Black race, male gender, and the presence of multiple comorbidities.” Why there is no information about black race in this study, only “nonwhite” ?

AU: We appreciate the reviewer’s important comments regarding race and ethnicity data. We have now reported the Black race separately in table 1, but other race remained as “other” due to low number. (see updated Table 1)

2) In results , it is said that “The No-AKI group had a higher comorbidity burden compared to AKI group”. What is the reason for that ? Is there any hypothesis?

AU: We appreciate the reviewer’s insightful question regarding the higher comorbidity burden in the No-AKI group and recognize the importance of discussing potential explanations.

(1) **Potential Explanation for Higher Comorbidity in the No-AKI Group**: One possible hypothesis is that patients with multiple chronic conditions may have had more frequent healthcare encounters, leading to earlier CKD diagnosis and medical management that potentially reduced the risk of AKI. In contrast, patients who developed AKI may have had fewer comorbidities initially but were exposed to acute risk factors (e.g., hospitalization, sepsis, nephrotoxic medications) that precipitated AKI events.

(2) **Impact of Healthcare Utilization on AKI Risk**: It is also possible that patients with a higher comorbidity burden were already under close clinical monitoring (e.g., for hypertension, diabetes, or cardiovascular disease), which might have led to preventive interventions that reduced the likelihood of AKI. Conversely, AKI patients may represent a population with more acute or transient risk factors rather than long-term chronic disease burden.

(3) **Discussion Revision to Address This Hypothesis**: To clarify this point, we have added the following explanation to the Discussion section: "One possible explanation is that patients with multiple chronic conditions may have been under closer clinical surveillance, leading to earlier CKD diagnosis and management, potentially reducing AKI risk. In contrast, AKI patients may have experienced more acute insults (e.g., hospitalization, sepsis, nephrotoxic exposure) rather than a higher baseline burden of chronic diseases.”

3) Finally, in discussion, item 4.2, 4.2: “Additionally, diagnoses leading to intravascular volume depletion, such as diarrhea, nausea with vomiting, and

dehydration, were uniquely associated with the HA-AKI cohort." This fact couldn´t be only due the lack of information, as it is easier to have this data when the patient is in hospital ?

AU: We acknowledge the reviewer's important point regarding the potential impact of data availability on the observed association between HA-AKI and volume depletion diagnoses.

(1) **Limitations of Capturing Volume Depletion in Outpatients**: Volume depletion-related diagnoses (e.g., diarrhea, dehydration, nausea with vomiting) are more frequently documented in hospital settings because patients are monitored more closely, and fluid balance is systematically assessed. In contrast, outpatient volume depletion may be underreported or inconsistently documented in electronic health records (EHR), particularly if it does not lead to hospitalization.
(2) **Data Availability & Potential Bias**: Our study relies on ICD-coded diagnoses, which may not fully capture the extent of volume depletion in non-hospitalized patients. The absence of outpatient fluid status measures (e.g., urine output, weight changes, serum electrolytes over time) limits our ability to comprehensively assess volume depletion risk in the CA-AKI group.
(3) **Revised Discussion to Acknowledge This Bias**: To address this potential limitation, we have updated section 4.2 in the Discussion: "The association between HA-AKI and volume depletion diagnoses (e.g., diarrhea, nausea with vomiting, dehydration) may be partially influenced by differences in data availability. These conditions are more easily captured in hospitalized patients, where fluid status is closely monitored, whereas outpatient volume depletion is likely underreported or inconsistently documented in EHR data. Future research with comprehensive outpatient laboratory and clinical data is needed to confirm these findings."

REVIEWER #3:

This manuscript is interesting; however, several edits are necessary to improve the research article:

1. The background section needs better organization, particularly in discussing AKI versus HA-AKI.

AU: The background section has been revised thoroughly in the revised version.

2. I recommend relocating the following passage to the methods section: "Variable clustering uncovers correlated clinical and demographic variables, revealing underlying patterns that influence disease progression. In contrast, network-based clustering constructs networks of variables, offering both visual and analytical insights to identify and group critical comorbidities and procedures that drive the progression from AKI to CKD. These two approaches complement each other by cross-referencing identified clusters, ensuring robust and accurate phenotyping."

AU: We appreciate the reviewer's suggestion and agree that this passage is more appropriate for the Methods section. The passage has been relocated to Methods section 2.7 (Hierarchical Diagnosis and Procedure Clustering) to better align with the description of our analytical approach.

3. While the study asserts as STROBE-compliant, I suggest providing supporting evidence for this.

AU: We appreciate the reviewer's suggestion and have taken steps to strengthen our reporting of STROBE compliance:

(1) **Flowchart Revision for STROBE Compliance**: The study flowchart has been revised to better align with STROBE guidelines for cohort studies, clearly outlining patient selection, inclusion/exclusion criteria, and follow-up timeframes. (Fig1)
(2) **Clarification of Study Design in Methods Section**: We have explicitly stated that this is a retrospective cohort study, following patients for three years from AKI to CKD diagnosis. To reinforce transparency, we included the following details in the Methods section: "This study adheres to the STROBE guidelines for observational research. The retrospective cohort design follows patients with AKI over three years to evaluate CKD progression. HA-AKI patients were defined as those who had AKI within 90 days of any inpatient hospitalization (identified by Current Procedural Terminology [CPT] codes: 99218-99239, 99251-99255, 99291, 99304-99307, 94002, G03378,1013699, and 1013659). CA-AKI patients were defined as those who had any AKI events, excluding HA-AKI, within three years prior to their CKD diagnosis. The primary outcome was the progression to CKD from two groups: hospital-acquired AKI (HA-AKI) and community-acquired AKI (CA-AKI)." (Subsections 2.1 and 2.4)

4. I find a mismatch between the study's stated aim and the actual work conducted. The authors assert that the aim is to understand the progression from AKI to CKD, yet the findings seem to focus on identifying risk profiles associated with CKD patients with and without AKI. This is further emphasized in the discussion section. For clarity and consistency, I recommend aligning the objective with what was accomplished in the study.

AU: We appreciate the reviewer's careful assessment and agree that the study objective should more accurately reflect the findings. We have revised the study objective in both abstract and introduction section in the revised version. To maintain consistency throughout the manuscript, we have adjusted the Discussion section to clearly frame our findings as an analysis of distinct CKD patient risk profiles associated with prior AKI events.

REVIEWER #4:

This research article is a commendable piece of scholarly work that contributes significantly to its field. The methodology adopted is robust, ensuring the reliability and validity of the findings. The literature review is comprehensive and provides a solid foundation for the research. The analysis is insightful, revealing new aspects that were previously unexplored.

The conclusions drawn are well-supported by the data, and the implications for future research are thought-provoking. The authors have done an excellent job of communicating complex ideas in a clear and accessible manner, making the article a valuable resource for both experts and novices. Overall, this article is a fine example of academic rigor and intellectual acumen, and it is sure to inspire further inquiry and discussion. Kudos to the authors for their hard work and dedication to advancing knowledge in their field.

AU: Thank you for your comments.

REVIEWER #5:

In this paper, Mohammad A. Al-Mamun and colleagues developed a new phenotyping framework for hybrid clustering (combine variable clustering and network-based clustering) to investigate the main risk factors that lead to chronic kidney disease (CKD). Specifically, the authors explored the risk of CKD after an acute-kidney injury (AKI) addressing multifactorial and complex progression from AKI to CDK, with the goal of improving clinical decision and patient care.
I believe the core idea of this study is innovative. However, several points are unclear, making it difficult to follow the authors' rationale and the results they presented. Consequently, the manuscript needs a deep revision. Below are my detailed comments:

ABSTRACT:
(a) The authors could add a bit more context to explain why understanding the AKI-to-CDK transition is so crucial, particularly in terms of public health impact.
(b) I suggest to report the number of patients used for the analysis after the filtering (58,606 patients) instead of the total dataset (90,602).

AU: The abstract was revised to state the following to improve impact fullness and clarity.

INTRODUCTION:
I strongly recommend discussing in more depth the limitations of existing clustering methods, while highlighting the advantages of the authors' proposed approach. In particular, the authors could explain why traditional patient-centric clustering techniques are inadequate for understanding this problem (highlighting their inability to capture temporal or interaction complexities).

AU: We would like to thank for these important suggestion. We have now revised the subsection 4.5.

It might also help to mention upfront how your two complementary methods (variable clustering and network-based clustering) were combined.

AU: Thank you for your comment. We have added a sentence in the 3rd paragraph of the introduction section

MATERIAL AND METHOD:
o **Please, define "ICD-9-CM" and "ICD-10-CM" (perhaps in "Definitions, Inclusion, and**

Exclusion criteria" section) for clarity. In addition, ensure consistency in how these are referenced throughout the text (for example "ICD-9-CM" or "ICD-CM 9").

AU: All has been changed to ICD-9-CM / ICD-10-CM; have highlighted the changes

Since the authors refer to the No-AKI cohort both as "No-AKI cohort" and "cohort 3", please clearly define the cohorts when introducing them. For example: "After applying the inclusion and exclusion criteria, we created three cohorts: HA-AKI (cohort 1), CA-AKI (cohort 2), and No-AKI (cohort 3)."

AU: Thank you for your comment. The cohort number has now been thoroughly changed to:

- Cohort 1-> HA-AKI
- Cohort 2-> CA-AKI
- Cohort3 -> No AKI

The language used in the "Statistical Analysis" section could be revised for improve readability.

AU: Thank you for your comment. The section *2.9 Statistical Analysis* has been revised to improve clarity (line 211-219)

Specify the statistical method used to compare network measures between AKI grups and No-AKI reference group.

AU: We have added it as a footnote in the updated Table 2.

In the sentence, "We binarized all diagnoses and procedures for each cohort based on their presence or absence prior to CKD diagnosis.", it would be useful to clarify if 1 was used for presence and 0 for absence, or vice versa.

AU: Thank you for your comment. We have made the revision in the sentence (line 148-149)

The process of developing the phenotypic network need clarification: Do the edges link diagnoses of disease recorded only within the same admission? From the cited literature, it seems edges link subsequent diagnoses of disease. Please explain this aspect in more detail. I highly recommend adding a Supplementary Figure illustrating the main components of the network.

AU: No, they are not from the same admission. We added a supplementary table for illustrating main component of the network.

o When discussing the observed-to-expected ratio (OER), consider explaining in

simple terms what it tells us—perhaps with a short example illustrating how co-occurrence affects the network.

AU: Thank you for your feedback. The explanation of OER is under subsection 2.6 comorbidity network modeling and subsection 3.2

o **The authors generated networks with undirected edges. Since the dataset spans multiple years (2010-2016), it is likely that certain diseases and/or procedures are consequences of others. Consider introducing temporal analysis to study how comorbidities and procedures evolve over time in AKI to CKD progression. The authors could model the sequence of disease occurrence and procedure over time, rather than treating them as static variables.**

AU: This is a great suggestion; We aim to develop a temporal/sequence algorithm in our future studies and currently, it is out of the scope of this study.

o **A survival analysis (e.g., Kaplan-Maier curves) could be used to estimate the time from AKI to CDK and to identify time-dependent risk factors.**

AU: Thank you for the suggestion, however we opted to develop the proposed method to understand the patient profiles from AKI to CKD progression.

RESULTS:

o In the final part of the introduction, the authors mention that one of the aims of this study is to identify disease-specific trajectories comparing HA-AKI and CA-AKI subpopulations. I recommend adding two additional columns to Table 1 to show the p-values for comparisons between HA-AKI and No-AKI, and between CA-AKI and No-AKI. Consequently, the main text should discuss the key differences between HA-AKI and CA-AKI.

AU: Thank you for the suggestion, however we prefer to compare the cases vs control (e.g., HA-AKI vs No-AKI and CA-AKI vs No-AKI)

o, Please check whether the figures are cited correctly in the text. For instance, Figure 2a seems to show degree measures, while Figure 2b shows betweenness centrality. However, the text refers only to Figure 2a, describing it as displaying betweenness centrality. Additionally, the caption for Figure 2 in the main text might be incorrect.

AU: We revised the explanation of figure 2 in the subsection 3.2.

o In supplementary figure 1, what do the colors represent? Please add a legend or

include this information in the text. In addition, insert that "Supplementary Figure 1A" refers to HA-AKI and so on.

AU: We added the description of the colors in the figure title (in the main text)
o In Supplementary Figure 1a, some edges appear thicker (for example, the link between nodes 82728 and 83540 in green).

o Are the edges weighted?

AU: Yes, we added the description of visualization in subsection 3.2.

o Since the authors commented in the main text the common nodes between the two AKI subtypes, I strongly suggest conducting a graph subtraction analysis. The authors could remove edges common to both networks, retaining only disease-specific paths.

AU: Thank you for the suggestion, however we were interested to know and visualize the patient profiles rather tracking them temporally. But we aim to do that in the future works.

o What is the Supplementary Figure 2a? Please, clarify.

AU: Thank you for catching this. It was a typo, we revised it.

o Ensure that Figure 3 is cited correctly in the main text.

AU: We have removed that figure, as it was redundant.

o The figures displaying the networks are unclear. I recommend either finding an alternative way to present the data or reducing the number of links to improve clarity.

AU: We have revised the adjusted some network parameters to generate clear network graphs for diagnosis and procedure among the three cohorts.

o After using the network to filter comorbidities and procedures, the authors could perform regression analysis using cohort membership (i.e., HA-AKI, CA-AKI, or No-AKI) as the outcome and the selected comorbidities or procedures (binary) as predictors. The resulting odds ratios would demonstrate how specific components act as risk factors.

AU: This is great suggestion; we have run regression analysis for the top 5 predictors to see how they demonstrate the risk of HA-AKI or CA-AKI. We have added another supplementary table. The relevant description is added in subsection 4.2.

DISCUSSION:

I suggest including references to studies that have used similar methodologies and explaining the advantages of the approach used here.

AU: We have cited multiple references on the related works and clarify how our proposed method has advantage over the traditional methods in the discussion section.

References

Lai CF, Liu JH, Tseng LJ, Tsao CH, Chou NK, Lin SL, et al. Unsupervised clustering identifies sub-phenotypes and reveals novel outcome predictors in patients with dialysis-requiring sepsis-associated acute kidney injury. Ann Med [Internet]. 2023 Dec 12 [cited 2024 Aug 11];55(1):2197290. Available from: https://www.tandfonline.com/doi/abs/10.1080/07853890.2023.2197290

Tan Y, Huang J, Zhuang J, Huang H, Tian M, Liu Y, et al. Fine-grained subphenotypes in acute kidney injury populations based on deep clustering: Derivation and interpretation. Int J Med Inform [Internet]. 2024 Jul [cited 2024 Aug 11];191:105553. Available from: https://pubmed.ncbi.nlm.nih.gov/39068892/

Raftery AE, Dean N. Variable Selection for Model-Based Clustering. J Am Stat Assoc [Internet]. 2006 Mar [cited 2024 Aug 11];101(473):168–78. Available from: https://www.tandfonline.com/doi/abs/10.1198/016214506000000113.

Chavent M, Genuer R, Saracco J. Combining clustering of variables and feature selection using random forests. Commun Stat Simul Comput [Internet]. 2021 [cited 2024 Aug 11];50(2):426–45. Available from: https://www.tandfonline.com/doi/abs/10.1080/03610918.2018.1563145

Horne E, Tibble H, Sheikh A, Tsanas A. Challenges of Clustering Multimodal Clinical Data: Review of Applications in Asthma Subtyping. JMIR Med Inform [Internet]. 2020 May 1 [cited 2025 Mar 19];8(5):e16452. Available from: https://pmc.ncbi.nlm.nih.gov/articles/PMC7290450/

Martins C, Neves B, Teixeira AS, Froes M, Sarmento P, Machado J, et al. Identifying subgroups in heart failure patients with multimorbidity by clustering and network analysis. BMC Med Inform Decis Mak [Internet]. 2024 Dec 1 [cited 2024 Aug 11];24(1). Available from: /pmc/articles/PMC11020914/

Gao CX, Dwyer D, Zhu Y, Smith CL, Du L, Filia KM, et al. An overview of clustering methods with guidelines for application in mental health research. Psychiatry Res. 2023 Sep 1;327:115265

Reviewer #6: This is a novel study with relevant findings that in some way question the existing assumptions regarding comorbidities that predispose to chronic kidney disease after acute kidney injury. The limitations of the study are well stated and the designs proposed to delve deeper into the subject are interesting.

AU: Thank you for your comment.
